# Default mode network activity in bipolar disorder

**DOI:** 10.1017/S2045796020000803

**Published:** 2020-09-08

**Authors:** Niccolò Zovetti, Maria Gloria Rossetti, Cinzia Perlini, Eleonora Maggioni, Pietro Bontempi, Marcella Bellani, Paolo Brambilla

**Affiliations:** 1 Department of Neurosciences, Biomedicine and Movement Sciences, Section of Psychiatry, University of Verona, Verona, Italy; 2 Department of Neuroscience and Mental Health, Fondazione IRCCS Ca’ Granda Ospedale Maggiore Policlinico, Milan, Italy; 3 Department of Neurosciences, Biomedicine and Movement Sciences, Section of Clinical Psychology, University of Verona, Verona, Italy; 4 Department of Computer Science, University of Verona, Verona, Italy; 5 Department of Neurosciences, Biomedicine and Movement Sciences, Section of Neurology, University of Verona, Verona, Italy; 6 Department of Pathophysiology and Transplantation, University of Milan, Milan, Italy

**Keywords:** Bipolar disorder, default mode network, resting-state fMRI, review

## Abstract

Since its discovery in 1997, the default mode network (DMN) and its components have been extensively studied in both healthy individuals and psychiatric patients. Several studies have investigated possible DMN alterations in specific mental conditions such as bipolar disorder (BD). In this review, we describe current evidence from resting-state functional magnetic resonance imaging studies with the aim to understand possible changes in the functioning of the DMN in BD. Overall, several types of analyses including seed-based and independent component have been conducted on heterogeneous groups of patients highlighting different results. Despite the differences, findings seem to indicate that BD is associated with alterations in both frontal and posterior DMN structures, mainly in the prefrontal, posterior cingulate and inferior parietal cortices. We conclude this review by suggesting possible future research directions.

## Introduction

Bipolar disorder (BD) is a complex mental disorder characterised by severe mood fluctuations associated with functional and cognitive disabilities that often persist throughout the entire life of patients (Solé *et al*., 2017). Specifically, BD is expressed with alternating phases of (i) mania, including abnormally elevated mood and increased energy levels and (ii) depression, consisting of severely depressed mood, anhedonia and insomnia/hypersomnia. Moreover, patients with BD show various degrees of neuro-cognitive impairments affecting several domains such as working memory, attention and executive control, all impacting on socio-occupational functioning (Robinson *et al*., 2006).

Considering the neurobiological nature of BD, it is crucial to study the structural and functional brain alterations associated with the disease, in order to provide novel insights for future neurobiological models and treatments. To date, however, neurobiological underpinnings of the disorder are still discussed, hindering the development of new pharmacological therapies and targeted rehabilitative interventions. Findings on brain structure in BD suggest ventricular, prefrontal and temporal abnormalities but remain largely inconsistent (Delvecchio *et al*., 2020). The most consistent findings on brain function in BD concern brain activation patterns during emotion processing, emotion regulation and reward processing tasks. Overall, BD seems to be marked by a generalised dysfunction of the dorsal and ventral systems, composed respectively of (i) the prefrontal cortex (PFC), anterior cingulate cortex (ACC) and the hippocampus and (ii) the insula, amygdala and ventral striatum (Chen *et al*., 2011; Phillips and Swartz, 2014). However, it is not clear whether the aforementioned networks are altered only during the execution of specific tasks or also at rest (*Bellani et al., 2020*).

Recently, greater attention has been given to the investigation of brain activity and connectivity in BD at rest, mostly by means of resting-state functional magnetic resonance imaging (rs-fMRI). Particularly, rs-fMRI is used to evaluate the functional interactions among remote neural systems that occur in a resting or task-negative condition, i.e. when an explicit task is not being performed. This technique provides information about intrinsic brain activity, not dependent on specific tasks or experimental settings. Most commonly, the brain functional connectivity (FC) at rest is studied by analysing the statistical dependencies among the blood oxygenation level-dependent (BOLD) signals from multiple brain regions, which reflect the spontaneous fluctuations in the oxygenation state of blood (determined by blood flow, blood volume and oxygen consumption) within them (Smitha *et al*., 2017).

The physiological substrates of resting-state activity are currently the object of speculation (Rosazza and Minati, 2011). By contrast, functional connectivity (FC) at rest (i.e. the statistical dependence among spatially remote neuronal systems (Friston *et al*., 1993)) is suggested to be not a simple ‘background’ activity but a complex dynamic involved in cognition and memory consolidation (Rosazza and Minati, 2011) consuming more than 20% of body's energy supporting neuronal signalling and functioning (Raichle and Mintun, 2006).

Rs-fMRI networks can be studied employing different analysis pipelines that primarily fall in two main categories, (1) seed-based analyses (SBA), which require a-priori definition of the brain network nodes, (2) blind-source separation techniques, which adopt an unbiased approach by grouping brain voxels/regions based on their latent time series (independent component analysis, ICA) (Joel *et al*., 2011). For details on these approaches see Smitha *et al*. (2017).

These techniques have enabled the discovery of multiple brain networks characterised by highly correlated spontaneous BOLD fluctuations. The most commonly studied networks include (i) the Salience Network (SN), composed of the ACC and the insula; (ii) the central-executive network (CEN) consisting of fronto-parietal regions and the posterior parietal cortex; (iii) the fronto-parietal network (FPN) that includes the cortex surrounding the intraparietal sulcus, the inferior parietal lobe, the dorsal motor cortex and the inferior frontal cortex; and (iv) the default-mode network (DMN) extending from the prefrontal medial cortex (mPFC) to the precuneus, posterior cingulate cortex (PCC) and inferior parietal cortex. The DMN is recognised as a key component of the brain functional architecture. The vmPFC composes the anterior part of the DMN and has been shown to modulate social behaviour, mood regulation, executive functioning and control processes (Damasio *et al*., 1994). The PCC, the precuneus and the lateral parietal cortices, which form the posterior DMN, are involved respectively in attentional regulation, consciousness, mental imagery and episodic memory processes (Cavanna, 2007; Leech and Sharp, 2013). All these functions have been found altered (at different extents) in psychotic disorders and BD (Clark *et al*., 2002; Torres *et al*., 2007). Moreover, the precuneus has been recently suggested to be part of a hippocampal-parietal network involved in learning and consolidation of everyday experiences. The global DMN is involved in multiple cognitive and affective functions such as emotional processing, self-referential mental activity, mind wandering, recollection of experiences and possibly exerts a modulatory role during attentional demanding tasks (Raichle, 2015). Since attentional deficits are a core feature of BD (Solé *et al*., 2017), studying the DMN in patients with BD might prove particularly beneficial to help to understand the neurobiological underpinnings of the disease.

Considering the potential role of the DMN in BD, the aim of this review is to describe current evidence of DMN connectivity in patients with BD.

The data search was conducted on the Pubmed, Scopus and Google Scholar databases. The following keywords were used for the search: ‘default mode network’ AND ‘bipolar disorder’. The inclusion criteria were: (i) original publication in a peer-reviewed journal between 2010 and 2020, (ii) English language, (iii) a diagnosis of BD for the patient groups and (iv) the analysis of the DMN activity patterns through rs-fMRI. After an initial screening 71 studies were identified of which 23 studies were included in the review.

Table 1 provides a description of methods and results of the included studies. In detail, 11 studies (47%) analysed the rs-fMRI activity through ICA, providing thus an unbiased overview of brain activity at rest as a whole (Joel *et al*., 2011), while ten studies (43%) adopted an SBA approach, which requires a-priori hypotheses on the regions of interest within the brain (Smitha *et al*., 2017). Finally, the remaining two studies analysed the rs-fMRI activity through a network analysis (Wang *et al*., 2016) and the Data Processing Assistant for Resting-State (DPARSF) analysis pipeline, a MATLAB toolbox based on the SBA principles which gives information about regional FC and homogeneity (Brady *et al*., 2017). Only six out of 23 studies enrolled unmedicated BD patients. Yet, only four out of 23 studies recruited mixed samples of BD patients based on the subtypes (i.e. BD-1, BD-2) or the clinical phases of the disorder (e.g. depression, mania, euthymia) (Martino *et al*., 2016; Rive *et al*., 2016; Brady *et al*., 2017; Zhong *et al*., 2019).

**Table 1.** Studies exploring the DMN with rs-fMRI in BD

Reference	Participants	Mean age (s.d.), Sex (m/f)	rsfMRI analysis	Medication	Results (BD *v*. HC)
Öngür et al. (2010)	17 BD; 14 SCZ; 15 HC	34.4 (12), 9 m/8f; 42.3 (9.5), 8 m/6f; 37.9 (9.5), 9 m/6f	ICA	Yes	↑ FC in the parietal cortex correlated with mania severity. ↓ FC in the ventral mPFC. ↓ FC in the mPFC (both SCZ and BD).
Khadka et al. (2013)	64 psychotic BD; 70 SCZ; 52 BP relatives; 70 SCZ relatives; 118 HC	35.1 (11.2), 35 m/29f; 37.4 (12.8), 43 m/27f; 40.6 (13) 18 m/34f; 40.8 (15.6), 26 m/44f; 36.4 (10.8), 55 m/63f	ICA	Yes	↓ FC in the posterior DMN (both SCZ and BD).
Ford et al. (2013)	15 BD-1; 15 MMD	20.6 (2.6), 10 m/5f; 19.8 (2.6), 4 m/11f	ICA	Yes	PCC activation inversely correlated with bipolarity index.
Marchand et al. (2014)	19 BD-2; 18HC	28.9 (7.7), 11 m/8f; 27.9 (4.9), 13 m/5f	SBA	Yes	↑ FC in the left medial dorsolateral FG. ↑ FC in the right medial FG.
Meda et al. (2014)	300 psychotic BD; 296 SCZ; 324 HC; 179 SCZ relatives; 206 BD relatives	36.7 (12.6), 112 m/188f; 34.9 (12.2), 199 m/97f; 35.2 (13.4), 180 m/144f; 43.8 (15.8), 53 m/126f; 39.8 (16.1), 74 m/132f	ICA	Yes	↓ FC in the mPFC (both SCZ and BD). ↓ FC in the PCC (both SCZ and BD). ↓ FC in the Precuneus (both SCZ and BD). Superior posterior DMN FC inversely correlated with negative symptoms on PANSS. Anterior and inferior posterior DMN FC inversely correlated with positive symptoms on PANSS.
Teng et al. (2014)	15 BD-1; 16 HC	42.6 (9.7), 10 m/5f; 43.4 (11.3), 11 m/5f	SBA	Yes	↓ FC between striatal regions and PCC.
Yip et al. (2014)	15 BD-2; 20 HC	23 (3.7), 8 m/7f; 22.2 (2.5), 10 m/10f	ICA	No	No differences in FC of the DMN were found.
Liu et al. (2015)	17 BD; 17 MDD	32.1 (8.5) 6 m/11f; 32.5 (9.7), 5 m/12f	ICA	Yes	(BD *v*. MDD) ↑ FC between mPFC and PCC. ↑ FC between right inferior parietal cortex and left hippocampus. ↓ FC between mPFC and hippocampus.
Magioncalda et al. (2015)	40 BD; 40 HC	44.6 (11.8), 13 m/27f; 43.9 (12.8), 14 m/26f	SBA	Yes	↓ FC between PACC and ACC (in depressed BD) ↓ FC between PACC and PCC (in manic BD)
Rey et al. (2016)	27 BD; 27 HC	42.6 (11.1), 12 m/15f; 40.8 (9.3), 12 m/15f	SBA	Yes	↑ FC between left amygdala and left ACC and PCC. ↓ FC between right amygdala and ACC (only non-euthymic BD). ↓ FC between PCC and ACC (only euthymic BD). ↑ FC between ACC and right vlPFC (only euthymic BD). ACC-PCC and ACC-Amygdala FC were modulated by rumination in non-euthymic patients. ACC-vlPFC FC was modulated by current mood states.
Martino et al. (2016)	20 depressed BD-1; 20 manic BD-1; 20 euthymic BD-1; 40 HC	44.7 (11.2), 18 m/42f; 46 (12), 8 m/12f; 43.1 (11), 8 m/12f; 43.9 (12.8), 14 m/26f	SBA	Yes	Balance between DMN and SMN (calculated with the Slow5 fSD DMN/SMN ratio) was significantly decreased in manic and increased in depressed patients. DMN/SMN ratio correlated positively with clinical scores of depressive and negatively with manic symptoms.
Goya-Maldonado et al. (2016)	20 depressed BD; 20 MDD; 20 HC	35.8 (10.2), 7 m/13f; 35.6 (10.4), 14 m/6f; 36.2 (11.3), 13 m/7f	ICA	Yes	↑ FC in the frontal-parietal regions (only BD patients). ↑ FC in the DMN (only MDD patients).
Wang et al. (2016)	37 BD-2; 37 HC	26.3 (8.5), 22 m/12f; 27 (8.6), 23 m/14f	Network analysis	No	↓ FC in the DMN (bilateral medial PFC, left precuneus, right PCC). ↓ FC right superior FG.
Rive et al. (2016)	23 remitted MDD; 26 remitted BD; 22 depressed MDD; 10 depressed BD	42.7 (10.4), 8 m/15f; 42.7 (10.7), 10 m/16f; 43.8 (8.8), 7 m/15f; 38.8 (11.1), 4 m/6f	ICA	No	MDD and BD depressed patients could be classified based on GM volumes and DMN FC with 69% accuracy. Prediction accuracy with other networks' FC did not exceed chance level.
Brady et al. (2017)	23 BD-1; 24 euthymic BD-1; 23 HC	27.7 (11.1), 17 m/6f; 30.9 (11.9), 16 m/8f; 29.7 (10.9), 16 m/7f	DPARSF	Yes	↑ FC between frontal and temporal lobes/precuneus (only in manic BD). ↑ FC in the mPFC. ↓ FC in bilateral, temporal and parietal regions (only in euthymic BD). In the DMN FC between frontal hubs and the rest of the DMN differentiated mood states and diagnosis.
Nguyen et al. (2017)	15 euthymic BD; 19 HC	44.3 (11.3), 1 m/14f; 46.9 (13.2), 5 m/14f	SBA	Yes	No group differences in the FC of the DMN were found. However, dynamic connectivity between mPFC and PCC was shown to be less variable in BD. Less variability in FC was associated with reduced processing speed and cognitive set-shifting.
Wang et al. (2017)	25 euthymic BD-2; 25 HC	28.5 (9.7), 9 m/16f; 28.6 (9.6), 8 m/17f	SBA	Yes	↓ FC between posterior cerebellum and DMN (PCC). ↓ FC left posterior cerebellum and right inferior parietal lobule.
Luo et al. (2018)	94 BD-2; 100 HC	27.1 (9.1), 51 m/43f; 28.3 (8.9), 45 m/55f	SBA	No	↓ FC in the dlPFC. ↓ FC in the mPFC.
Chen et al. (2019)	90 BD-2; 100 HC	26.7 (8.7), 48 m/42f; 28.3 (9.), 45 m/55f	SBA	No	↑ FC between cerebellum and bilateral precuneus. ↓ FC between cerebellum and mPFC and MFG.
Qiu et al. (2019)	96 BD-2; 100 HC	27.3 (9.2), 52 m/44f; 8.3 (9), 45 m/55f	SBA	Yes	↓ FC between PCC and mPFC and precuneus.
Zhong et al. (2019)	25 psychotic BD; 23 non-psychotic BD; 18 HC	15.2 (1.5), 11 m/14f; 14.7 (2.1), 12 m/11f; 14.1 (1.6), 7 m/11f	ICA	Yes	↓ FC in the ACC and mPFC (psychotic BD *v.* HC). ↑ FC in the PCC (psychotic BD *v*. HC). ↓ FC in the mPFC (psychotic BD *v*. non-psychotic BD).
Wang et al. (2020)	51 depressed BD-2; 51 MDD; 52 HC	26.3 (8.7), 27 m/24f; 28.4 (8.4), 29 m/22f; 29.7 (11.1), 32 m/20f	ICA	No	↓ FC dynamic FC variability between posterior DMN and central executive network (both MDD and BD patients).
Bellani et al. (2020)	15 euthymic BD; 27 HC	40.2 (13.5), 5 m/10f; 37 (10.6), 6 m/21f;	ICA	Yes	↑ FC connectivity between motor area network and the DMN as a whole partially overlapping with the FPN.

↑, increased; ↓, decreased; ALFF, amplitude low frequency fluctuations; BD, bipolar disorder patients; BD-1, bipolar disorder type 1; BD-2, bipolar disorder type 2; dlPFC, dorsolateral PFC; DMN, default mode network; DPARSF, Data Processing Assistant for Resting-State fMRI; FC, functional connectivity; FG, frontal gyrus; GM, grey matter; HC, healthy controls; ICA, independent component analysis; MDD, patients with major depression; PFC, prefrontal cortex; MFG, medial frontal gyrus; mPFC, medial PFC; PACC, perigenual anterior cingulate cortex; PANSS, positive and negative symptoms scale; PCC, posterior cingulate cortex; ReHO, regional homogeneity; SBA, seed based analysis; SCZ, schizophrenic patients; SMN, sensorimotor network.

Overall, findings from the reviewed studies suggest that BD patients compared to Healthy Controls (HC) are characterised by marked functional alterations of both anterior and posterior hubs of the DMN, including the PFC, PCC, precuneus and inferior parietal cortex. These alterations are mostly mixed across studies, consisting of both hypo and hyper-connectivity of the anterior DMN at rest (Table 1).

Specifically, 14 studies (60%) showed aberrant functional activity in the posterior hubs of the DMN, of which five in the precuneus (21%) and 12 in the PCC (52%). These alterations are mostly hypo-activations of BD patients when compared with HC. Specifically, most of the studies showed FC reductions in BD compared to HC within these regions (Khadka *et al*., 2013; Meda *et al*., 2014; Teng *et al*., 2014; Magioncalda *et al*., 2015; Rey *et al*., 2016; Wang *et al*., 2016, 2017; Luo *et al*., 2018; Qiu *et al*., 2019). However, few recent studies found also hyper-connectivity between PCC, the mPFC and the insula (Liu *et al*., 2015). A single study by Zhong and colleagues differentiated between psychotic and non-psychotic BD and showed an increase in the FC of the PCC only in psychotic BD *v*. HC, possibly indicating specificity of the PCC alterations for the psychotic conditions (Zhong *et al*., 2019). Moreover, a study by Ford and colleagues (2013) found a significant negative correlation between FC in the PCC and a so-called ‘bipolarity index’ based on five key domains of the disorder: signs and symptoms, age of onset, course of illness, response to treatment and family history (Ford *et al*., 2013). Even though the authors do not speculate about this specific finding, the PCC has been previously found to be a key region in several cognitive processes such as monitoring of awareness, arousal, internal thought and attention control (Leech and Sharp, 2013). Of note, a single study performed a machine learning discrimination analysis based on the DMN FC between depressed BD patients and patients suffering from major depression (MDD), reaching a 69% accuracy (Rive *et al*., 2016). This result suggests that specific alterations of the DMN could be considered an endophenotype of BD. Partially in line with this hypothesis, two studies investigated FC of the DMN in both BD and schizophrenic (SCZ) patients and their close relatives (Khadka *et al*., 2013; Meda *et al*., 2014). Notably, only the study by Khadka and colleagues found alterations in the frontal hub of the DMN in both BD patients and their relatives (Khadka *et al*., 2013).

Some studies show the presence of altered connectivity in BD patients, specifically between the posterior part of the DMN and the anterior cingulate cortex (ACC) suggesting a possible relationship between the two regions (Magioncalda *et al*., 2015; Rey *et al*., 2016). Specifically, Magioncalda and colleagues (2015) found that in BD patients, alterations in the FC between the frontal and posterior hubs of the cingulate cortex can lead to excessive focusing on external contents and, ultimately, manic phases (Magioncalda *et al*., 2015).

Only two studies found no alterations in the DMN of BD patients (Yip *et al*., 2014; Nguyen *et al*., 2017). Nguyen and colleagues (2017) failed to find DMN alterations of BD patients, however, they found a reduction in the variability of the dynamic connectivity between the medial PFC and PCC associated with impaired processing speed (Nguyen *et al*., 2017). Unlike the standard rs-fMRI analysis pipelines, which estimate the average brain connectivity over time, dynamic connectivity methods allow to analyse how the FC between two or more brain regions changes during the entire scan duration obtaining useful information about the dynamic variability of the brain regional synchronization (Rashid *et al*., 2014). This result suggests the importance of considering not only the average FC over time but also the dynamic interplay between brain regions. Goya-Maldonado and colleagues (2016) found alterations in the DMN of patients suffering from major depression but not in the BD group. However, they found alterations in the FPN, a network partially overlapping with the DMN itself (Goya-Maldonado *et al*., 2016; Bellani *et al*., 2020). Lastly, also Yip *et al*. (2014)did not find any alteration of the FC of the DMN in BD(. However, the authors recruited only young BD-II patients (mean age 23.07 ± 3.73) without any history of medication. The authors suggest that the absence of alterations within the DMN may be due to specificity of the recruited group (e.g. diagnosis, last clinical phase) or on the absence of medication.

To conclude, current evidence from rs-fMRI studies suggests that BD patients show FC alterations (i.e. hypo and hyper-connectivity) compared to HC in both frontal and posterior hubs of the DMN. In particular, the evidence to date suggests that BD patients compared to HC have altered FC of the DMN in a number of regions including the PFC, the ACC and PCC and the precuneus. However, only a paucity of studies has been conducted and evidence is still sparse. The inconsistency of findings may be due (in part) to methodological issues such as heterogeneous analysis pipelines (e.g. ICA *v.* SBA *v.* regional homogeneity analysis), different BD populations (e.g. euthymic, BD-I, BD-II) or the lack of control for confounders (e.g. pharmacological treatment) that may have influenced the results (Vargas *et al*., 2013; Martino *et al*., 2016). Moreover, a recent study by Bellani *et al*. (2020), examining the resting state activations of a group of euthymic BD patients and HC, found alterations in the DMN only when examining between network connectivity, reporting no differences between the two groups in within-network functioning. The result suggests the importance of examining both within and between-network connectivity to achieve a global understanding of the BD euthymic condition.

Detailed neurophysiological correlates of resting-state activity are currently the object of speculation and only specific analysis techniques (i.e. dynamic functional connectivity) can account for the complex dynamics in the interactions between brain regions as suggested by some studies (Brady *et al*., 2017; Wang *et al*., 2020). Furthermore, since the BOLD contrast reflects hemodynamic/metabolic processes in the brain, the interpretation of rs-fMRI findings in terms of the underlying neuronal activity is not straightforward and would require the integration of electrophysiological information (Maggioni *et al*., 2016). Despite these limits, rs-fMRI studies allow the whole-brain mapping of the functional connectomes of specific pathologies such as the BD and provide new data for the development of neurobiological models and theories.

In view of the recent conceptualisation of BD in terms of neural circuitry, future studies should consider a multimodal perspective in order to (1) investigate the complex link between structure and function in the DMN and other networks involved in BD pathophysiology, (2) identify neurodevelopmental and maturation trajectories of FC patterns in relation to the natural course of the disease and in response to different therapeutic strategies, (3) disentangle the contributions of genetics, environment and their interaction to the large-scale functional brain network abnormalities underlying BD symptomatology.

## Data

The data that support the findings of this study (search query) are available on request from the corresponding author.
